# Supplementary material for: Carbon Fibers Prepared via Solution Plasma-Generated Seeds
Source: Materials (Basel). 2023 Jan 17;16(3):906. doi: 10.3390/ma16030906 (PMC9918063; doi:10.3390/ma16030906)
Supplement: Supplementary file 1 [file materials-16-00906-s001.zip › materials-2148388-supplementary.pdf]

SUPPLEMENTARY INFORMATION

Figure S1. Nickel particles and Fiber diameter

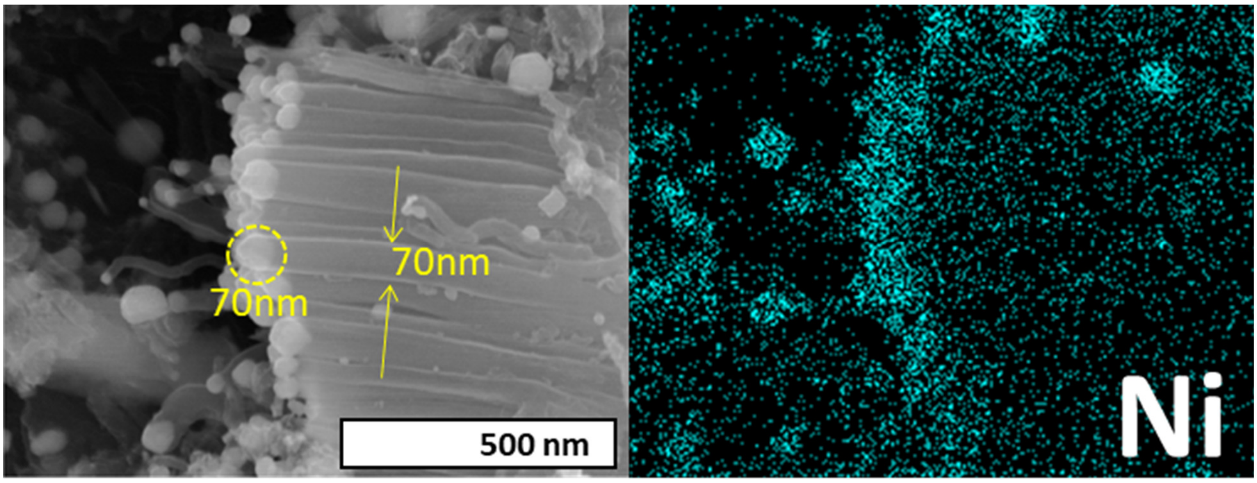

Figure S1 shows a SEM-EDX image of a dichlorobenzene grown bundle in which the Ni metal particle size determines the diameter of the fiber.

Figure S2. FTIR of carbon fibers grown by different precursors.

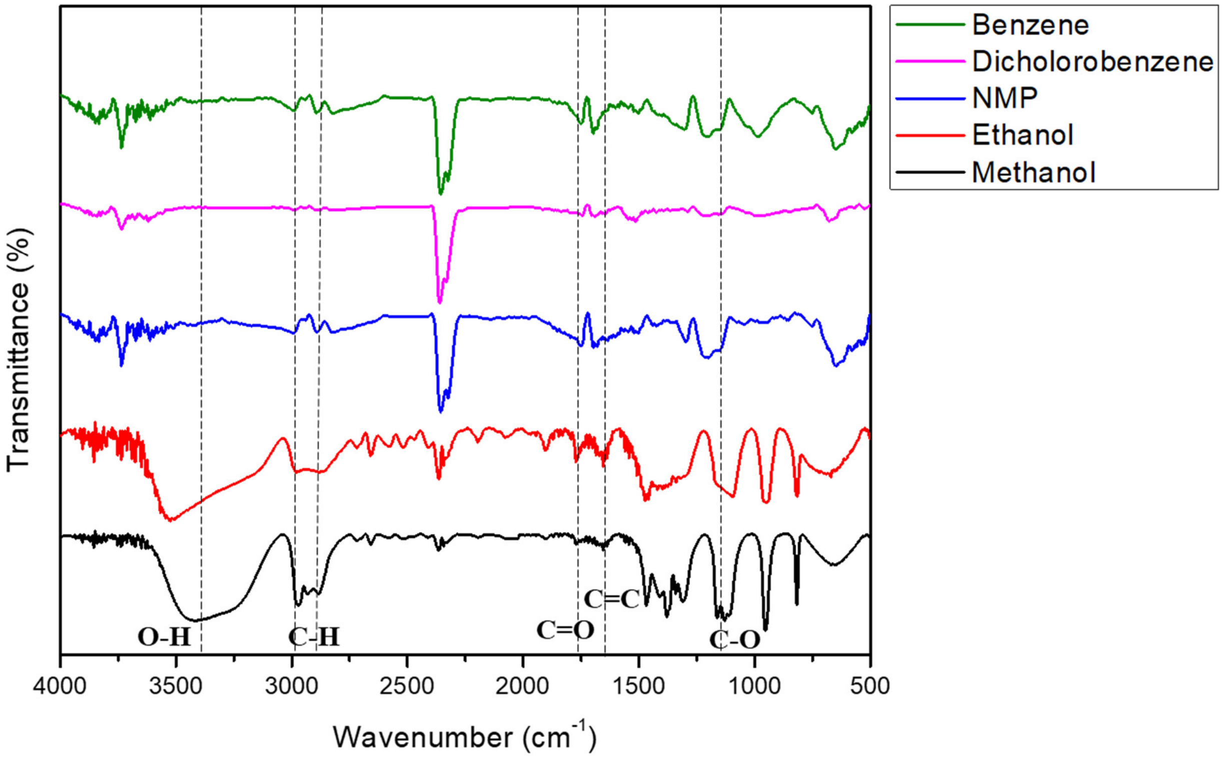

Figure S2 shows the FTIR of carbon fibers grown by different precursors. The bands observed at around 3400, 2900, 1750, 1640 and 1100 cm^{-1}, correspond to the functional groups O-H, C-H, C=O, C=C and C-O respectively.
